# A case of pure apraxia of speech after left hemisphere stroke: behavioral findings and neural correlates

**DOI:** 10.3389/fneur.2023.1187399

**Published:** 2023-07-27

**Authors:** Alexis L. Pracar, Maria V. Ivanova, Amber Richardson, Nina F. Dronkers

**Affiliations:** ^1^Department of Psychology, University of California, Berkeley, Berkeley, CA, United States; ^2^VA Northern California Health Care System, Martinez, CA, United States; ^3^Department of Neurology, University of California, Davis, Davis, CA, United States

**Keywords:** aphasia, apraxia of speech, insula, Broca’s area, speech production

## Abstract

**Introduction:**

Apraxia of speech (AOS) is a motor speech disorder impairing the coordination of complex articulatory movements needed to produce speech. AOS typically co-occurs with a non-fluent aphasia, or language disorder, making it challenging to determine the specific brain structures that cause AOS. Cases of pure AOS without aphasia are rare but offer the best window into the neural correlates that support articulatory planning. The goal of the current study was to explore patterns of apraxic speech errors and their underlying neural correlates in a case of pure AOS.

**Methods:**

A 67-year-old right-handed man presented with severe AOS resulting from a fronto-insular lesion caused by an ischemic stroke. The participant’s speech and language were evaluated at 1-, 3- and 12-months post-onset. High resolution structural MRI, including diffusion weighted imaging, was acquired at 12 months post-onset.

**Results:**

At the first assessment, the participant made minor errors on the Comprehensive Aphasia Test, demonstrating mild deficits in writing, auditory comprehension, and repetition. By the second assessment, he no longer had aphasia. On the Motor Speech Evaluation, the severity of his AOS was initially rated as 5 (out of 7) and improved to a score of 4 by the second visit, likely due to training by his SLP at the time to slow his speech. Structural MRI data showed a fronto-insular lesion encompassing the superior precentral gyrus of the insula and portions of the inferior and middle frontal gyri and precentral gyrus. Tractography derived from diffusion MRI showed partial damage to the frontal aslant tract and arcuate fasciculus along the white matter projections to the insula.

**Discussion:**

This pure case of severe AOS without aphasia affords a unique window into the behavioral and neural mechanisms of this motor speech disorder. The current findings support previous observations that AOS and aphasia are dissociable and confirm a role for the precentral gyrus of the insula and BA44, as well as underlying white matter in supporting the coordination of complex articulatory movements. Additionally, other regions including the precentral gyrus, Broca’s area, and Area 55b are discussed regarding their potential role in successful speech production.

## 1. Introduction

Acquired apraxia of speech (AOS) is a motor speech disorder that impairs the coordination of complex articulatory movements needed to produce speech. It is characterized by slow speech rate, segmentation of syllables, sound distortions, distorted substitutions, trial-and-error articulatory movements, and particular difficulty with long and complex utterances (1). AOS is often accompanied by a language disorder known as aphasia but is considered a unique motor speech disorder. Errors from AOS arise from poor coordination of the speech articulators, whereas errors from aphasia are language-based. Cases of pure AOS bear witness to the distinction between motor speech and language. They offer a view into the unique neural correlates and fiber pathways that support the coordination of articulatory speech movements. AOS is often thought to simply be a part of Broca’s aphasia, but pure cases demonstrate that this is not the case. The goal of the current study was to explore patterns of apraxic speech errors and underlying neural correlates in a case of pure AOS across the first-year post-stroke. Here we provide motor speech, language, and neuroradiological findings, including white matter tractography.

A 67-year-old right-handed male presented with a left middle cerebral artery stroke possibly caused by previously undiagnosed atrial fibrillation. The participant had a history of chronic hypertension. Immediately following the stroke, the participant exhibited mutism and dysphagia, which quickly resolved leaving only a very mild aphasia and severe motor AOS. Right-handed weakness and clumsiness were also initially present but dissipated in the first 3 weeks post-onset. The participant received speech therapy 1 to 2 times per week for 45-min sessions in the first-year post-stroke. The participant’s writing was initially impaired by mild agrammatism. Reading comprehension and memory were intact. Severe difficulty with speech production persisted resulting in phonemic paraphasic errors and trouble with articulation, indicating continuing motor AOS. Within the first few weeks, the participant reported improvements in his mild language difficulties. By 3 months post-onset, the aphasia had completely resolved on standardized tests, leaving only a persisting AOS. Currently, the participant has returned to work in his prior profession though with modifications, considering his speech difficulty. The participant was given quantitative speech and language assessments at 1-, 3- and 12-months post-onset and completed structural and diffusion scans at 12 months post-onset. Behavioral and neuroimaging data are described in detail below.

## 2. Background

### 2.1. Apraxia of speech

AOS does not entirely deprive someone of the ability to produce speech sounds, but rather, the ability to coordinate the movements of the lips, tongue, velum, and larynx so they can shift rapidly to correctly produce the desired phonemes. Accordingly, the errors produced tend to approximate the target word but are inconsistent from trial to trial (e.g., /winson/,/winton/ for ‘winston’). Articulatory groping for the correct pronunciation is typical, accompanied by a disruption in prosody and rate of speech (1–3). AOS errors are especially vivid during the rapid repetition of complex sound sequences or with words and phrases that require rapid transitions between places of articulation, as in ‘geography.’ Consonant clusters are most difficult (e.g., the /gl/ in ‘glisten’) because, without vowels to extend the transition (as in ‘guppy’), the time to reposition the articulators for each consonant is greatly reduced.

Though interdependent for communication, motor speech and language processes are different. It is possible for someone to perform perfectly on language production and comprehension measures and still be diagnosed with a motor-speech disorder, such as dysarthria or AOS (4). Dysarthria is a motor disorder characterized by consistent speech distortions caused by muscle weakness. On the other hand, AOS causes inconsistent articulatory errors with no muscle weakness (3). Patients with AOS do not struggle with utterance formulation as do those with aphasia, nor do they struggle with the execution of motor plans as do those with dysarthria (4). AOS is believed to arise at a stage of speech production that occurs after utterance formulation (i.e., word-finding, sentence structuring), at the point when the articulators need to be coordinated for speech production, but before they are put into action.

Proper diagnosis of AOS requires a detailed evaluation by a clinician trained in the difference between AOS and other speech and language disorders, as there is a great deal of confusion about what AOS is and what it is not. There have been at least 23 different labels created to describe speech production problems, AOS being just one of them (2). AOS has erroneously become an umbrella term for other speech production disorders, though Darley suggested clear distinctions between AOS, dysarthria, and other motor speech disorders (4).

In the first days post-stroke, AOS can be transient (5), presumably due to edematous effects on the brain, which can resolve quickly (6). Some strokes cause persistent or chronic AOS, whereas in other cases, it can emerge due to neurodegeneration, such as in primary progressive aphasia (PPA), where it is often the first symptom in the non-fluent variant (2, 7, 8). Primary progressive apraxia of speech (PPAOS) occurs when apraxia of speech is progressive and the first or sole clinical symptom preceding the diagnosis of a neurodegenerative disease (9). Because AOS is most commonly caused by a stroke in the distribution of the middle cerebral artery (MCA), it is often accompanied by non-fluent aphasias, such as Broca’s or Global aphasia. The frequent co-occurrence of aphasia and AOS makes it challenging to determine specific brain regions that cause AOS.

### 2.2. Neural correlates of AOS

Studies regarding the neural correlates of AOS have produced varying results as there is a dearth of pure cases reported in the literature. Originally, the left posterior inferior frontal gyrus, including the region commonly known as Broca’s area was suspected to be responsible for speech articulation [e.g., (10, 11)]. Hillis and colleagues used diffusion-weighted imaging and perfusion-weighted imaging in an acute cohort of 40 patients with insular damage and 40 without. They determined a relationship between Broca’s area and AOS. Another study in chronic cases of aphasia was performed by Richardson and colleagues, analyzing 26 cases of left hemisphere stroke resulting in a classification of aphasia with apraxia of speech. Among those, 15 had Broca’s aphasia. In 26/26 patients with AOS, they found maximal overlap in the middle insula. However, stepwise regression with damage to four cortical regions of interest (the anterior insula, the posterior insula, Broca’s area pars opercularis, and Broca’s areas pars triangularis) as predictors of AOS suggested that damage to Broca’s area (pars opercularis) was most predictive of AOS.

Several large group studies have demonstrated that damage specifically to the left superior precentral gyrus of the insula (SPGI), a very small subregion of the insula, can lead to AOS (2, 12, 13). Dronkers (13) evaluated 44 patients with left hemisphere stroke, 25 of whom were diagnosed with AOS, implicating the left SPGI by a double dissociation; all cases with AOS involved damage to the SPGI whereas cases without AOS completely spared the SPGI. Ogar et al. (2) also noted the SPGI in an analysis of 18 patients with AOS and 8 without AOS, with more widespread lesions increasing the severity of symptoms. One criticism of this conclusion is that the SPGI is a commonly infarcted region in MCA stroke, and thus an overlap of MCA lesions would naturally converge in this area. However, patients without AOS who also had MCA strokes did not have convergent lesions in the SPGI, but rather, spared the area completely. In addition, Baldo and colleagues also suggested the SPGI was key for complex articulation in a cohort of 33 patients using voxel-based lesion-symptom mapping (VLSM), a statistically-based method of lesion analysis that accounts for frequency of damage, such as common areas of infarction (12). More recently, Tomaiuolo et al. (14) presented four cases of pure apraxia of speech, defined as cases of AOS with no aphasia or orofacial weakness. These cases of pure AOS had lesions located in the left precentral gyrus of the insula, and all cases spared Broca’s area. Finally, Oliveira and colleagues reported a case of persistent developmental AOS, beginning in early childhood, caused by a focal lesion to the SPGI (15).

Other groups suggest the role of the left precentral gyrus of the lateral motor cortex in AOS. In a study of 7 patients with pure AOS and 15 patients with AOS and concomitant aphasia, Itabashi et al. (16) found that the left precentral gyrus was the most common region associated with AOS (16). However, Itabashi et al. used a qualitative diagnosis for AOS, with no quantitative measures of errors, based on perceptual observations of conversation and repetition. Though this method is frequently used for AOS diagnosis, it does leave us without a way to compare these cases quantitatively with other cases that are reported in the literature. Graff-Radford et al. (17) analyzed 5 acute cases of pure AOS and found the greatest area of lesion overlap centered around the left premotor and motor cortices, however, 4 of 5 of the cases of “pure AOS” also had orofacial weakness. In patients with PPAOS, structural MRI scans have revealed atrophy in the lateral premotor and supplementary motor area (SMA) (9, 18, 19). Interestingly, in PPAOS, both left and right hemispheres can be affected (20). It is also reported that severity of PPAOS symptoms worsens if atrophy and patterns of hypometabolism spread to inferior frontal regions, primary motor cortex and the brainstem. A recent case study by Utianski and Josephs (21) of a woman with PPAOS showed hypometabolism in bilateral premotor and supplementary motor areas. However, the left hemisphere was slightly more affected, with hypometabolism affecting the left middle and inferior frontal cortices (21). Finally, a single case of AOS was reported after neurosurgical resection of an astrocytoma in the posterior middle frontal gyrus [Human Connectome Project Area 55b; (22)], adjacent to the left dorsal premotor cortex (23).

Speech and language arise from networks of cortical areas connected by fiber bundles. Fiber tract lesions are sufficient to cause production problems. The frontal aslant tract (FAT) is a fiber tract that connects the superior and inferior frontal gyri. Specifically, it runs from the supplementary motor area to the pars opercularis of the inferior frontal gyrus (24, 25). Since the FAT connects the inferior frontal gyrus with pre-supplementary and supplementary motor areas, it may have a role in motor speech planning and control and has been associated with AOS symptoms (26). In a study of 52 patients with chronic aphasia and concomitant AOS, Chenausky and colleagues identify lesions to the insula and to the dorsal arcuate fasciculus (AF) as being most predictive of AOS (27). Valls Carbo et al. (28) specifically examined the supplementary motor area and its involvement in AOS and agrammatism for cases of PPAOS. AOS is often the first sign of PPA in the non-fluent variant (7) and can persist in the form of PPAOS in other cases (9). Valls Carbo et al. (28) showed abnormal patterns of diffusion in 36 cases of AOS prior to the onset of aphasia (thus classified as PPAOS) in the supplementary motor area (SMA) fibers. Specifically, the SMA commissural fibers, and white matter projections from the SMA to inferior frontal cortex (via the FAT), U-fibers to the motor cortex, and to the basal ganglia may contribute to speech production (28).

Several factors contribute to the lack of consensus regarding the neural correlates of AOS in the previous literature. First, the criteria for the diagnosis of AOS are not uniform across studies. Though Darley and colleagues made the distinction between AOS and dysarthria (4), AOS and dysarthria are still frequently confused. The presence of other concomitant disorders (such aphasia and dysarthria) also impacts the ability to diagnose AOS with certainty. The time point of assessment (acute versus chronic) and the neuroimaging techniques used to determine lesion site (e.g., MRI, diffusion-tensor imaging, perfusion-weighted imaging) may also impact the reliability of a given study. Pure cases of AOS can help to guide future large group studies, as they are not confounded by co-occurring disorders.

The goal of the current study was to describe the injury to cortical regions and surrounding white matter that resulted in a pure case of AOS, as characterized by detailed speech and language assessments.

## 3. Methods

### 3.1. Behavioral methods

#### 3.1.1. Quantitative speech and language measures

The participant underwent a complete speech and language evaluation at 1-, 3- and 12-months post-onset to track both language outcomes and changes in language abilities across the first-year post-stroke. All tests were administered by two individuals highly trained in the assessment of speech and language disorders. Additionally, a certified speech and language pathologist (AR) was consulted at 1-year post-onset to confirm the ongoing presence of AOS (see Supplementary Appendix). In the current study, the following comprehensive testing protocol was used: the Comprehensive Aphasia Test (29) and the Western Aphasia Battery-Revised (WAB-R) (30) were administered as overall language assessments; The Curtiss-Yamada Comprehensive Language Evaluation-Receptive (CYCLE-R) (31) to evaluate sentence comprehension at varying levels of complexity; the Action and Object naming subtests from the Northwestern Naming Battery (32) for the evaluation of production deficits at the word level; the Sentence Production Priming Subtest from the Northwestern Assessment of Verbs and Sentences (32) for evaluating production deficits at the sentence level. The entire testing protocol was administered in 2 to 3 sessions, between 60 and 90 min each, including breaks.

#### 3.1.2. Comprehensive aphasia test (CAT)

This test includes eight language subtests: comprehension of spoken language, comprehension of written language, repetition, naming, spoken picture description, reading, writing, and written picture description (29). The test provides a convenient conversion to T-scores for each subtest and enables a direct comparison across language modalities.

#### 3.1.3. Western aphasia battery-revised (WAB-R)

The WAB is also a test of general language abilities in aphasia but was used here as an independent assessment of overall severity of language deficits (30). The participant was assessed with tests of spontaneous speech, auditory verbal comprehension, repetition, and naming. Scores from these subtests comprise the WAB Aphasia Quotient (AQ), a general measure of aphasia severity.

#### 3.1.4. Curtiss-Yamada comprehensive language evaluation-receptive (CYCLE-R

This test is used to evaluate sentence-level comprehension (31). In this test, participants hear a sentence and are presented with three- or four-picture arrays, in which they must select the target picture corresponding to the sentence. The test is particularly sensitive to deficits in syntactic processing, as it uses a very limited vocabulary while assessing comprehension of sentence structures with varying levels of difficulty ranging from simple sentences to complex multi-clause relatives.

#### 3.1.5. Northwestern naming battery (NNB) and assessment of verbs and sentences (NAVS)

From the NNB, subtests on action and object naming were used that have been matched on lexical frequency and length (32). Naming deficits are the most prevalent language deficits across severity levels and types of aphasia. The sentence production priming subtest of the NAVS is used to assess sentence production in canonical and non-canonical forms. In this task, participants hear a sentence and are instructed to produce a sentence like the one they heard but that now describes a different picture they see before them. This method of assessment was chosen in addition to more traditional picture description tasks as it allowed us to directly probe the production of various sentence types including Actives, Subject Relatives, Subject WH-questions, Passives, Object Relatives, and Object WH-questions.

#### 3.1.6. Motor speech evaluation (MSE)

The MSE is used to assess the motor speech deficits of dysarthria and AOS (3). The MSE elicits speech samples with such tasks as vowel prolongation; repetition of syllables, words, and phrases; oral reading; repetition, and picture description. A score of 0 indicates the absence of a deficit, while a score of 1 through 7 reflects the level of severity for each of the two disorders. The MSE uses eight subtests to characterize motor speech deficits:

1. **Vowel Prolongation**: The participant is asked to take a medium-sized breath and produce the prolonged vowel “ah” for as long as possible. This subtest indicates the presence of adequate breath support for speech production. The examiner listens for tremors and fluctuations in volume.
2. **Sequential Diadochokinesis**: The participant produces multiple repetitions of one-syllable strings (“*puh puh puh*…,” “*tuh tuh tuh*…,” and “*kuh kuh kuh*…”) to assure each sound can be produced and to determine maximum repetition rate.
3. **Alternating Diadochokinesis**^1^: The participant combines the same 3 sounds to repeatedly produce the string, “*puh tuh kuh…*,” as rapidly as they can manage. This task requires transitions between different points of articulation (bilabial, apico-alveolar, and velar).
4. **Single Repetition of Multisyllabic Words**: The participant produces single repetitions of three multisyllabic words (‘gingerbread’, ‘snowman’, and ‘television’) after the examiner provides a model. Some of these words contain consonant clusters.
5. **Multiple Repetitions of Multisyllabic Words** (see text footnote 1): The participant repeats three polysyllabic words five times each (‘artillery,’ ‘impossibility,’ and ‘catastrophe’). These words include consonant clusters and require rapid travel between multiple places of articulation in the mouth.
6. **Single Repetition of Monosyllabic Words**: The participant produces single repetitions of monosyllabic words (e.g., ‘nine,’ ‘judge’) after the examiner provides a model. Each word starts and ends with the same consonant, requiring minimal travel between places of articulation in the mouth and throat.
7. **Repetition of Sentences** (see text footnote 1): The participant repeats sentences comprised of high frequency and low frequency words (i.e., ‘In the summer they sell vegetables,’ ‘Arthur was an oozy, oily sneak’).
8. **Reading of “Grandfather Passage”** (see text footnote 1): The participant reads a short paragraph designed to contain the sounds most commonly produced by English speakers. This test is used to compare speech during reading to speech on repetition.

### 3.2. Neuroimaging methods

#### 3.2.1. Structural data

High-resolution structural scans for anatomical localization were acquired using a 3D T1w MPRAGE (magnetization-prepared rapid gradient echo) protocol with 1 mm^3^ isotropic resolution (TR/TE/TI = 2,300 / 2.96 / 900 ms; flip angle = 9°; FOV = 256 mm; imaging matrix = 256 × 256; acquisition time = 5.12 min) based on the ADNI-3 protocol (33). Also, FLAIR (TR/TE/TI = 4800/442/1650 ms; FOV = 256 mm; imaging matrix = 256 × 256; acquisition time = 4.21 min) and fast spin echo T2-weighted (TR/TE = 3200/408 ms; FOV = 256 mm; imaging matrix = 256 × 256; acquisition time = 4.08 min) images both with 1 mm^3^ isotropic resolution were acquired to confirm the location of the lesions and to aid in tissue normalization. The participant’s lesion was manually reconstructed with methods that we have used successfully in our previous studies (13, 34, 35) using ITK-SNAP software (36) and blindly reviewed by our neurology consultant (RTK).

Next, the ANTs toolbox (37) was used to perform brain extraction on the structural T1s and transform these images to MNI152 space. The transformation to MNI space consisted of an initial rigid plus affine transformation followed by a diffeomorphic “SyN” transformation, while cost-function masking the lesion. We used the MNI152NLin2009cAsym version of the MNI image as the moving image, and the T1 as the reference image (38). The inverse transformation was then applied to bring the T1 and the lesion mask into MNI space. This was done to obtain the most accurate overlap with atlas-based regions of interest.

Standard regions of interest were first taken from the Harvard Oxford Atlas. Notably, the atlas contained specific ROIs for BA44/45, the precentral gyrus on the motor strip, and a ROI for the entire insula. Additionally, ROIs were taken from the Brainnetome atlas (39), to divide the larger Harvard Oxford regions into smaller parcellations. Two additional ROIs were added based on the previous literature that suggested a role for them in AOS. First, a specific ROI for the SPGI was segmented based on Dronkers (13) and second, a ROI for area 55b (23) was added based on the Human Connectome Project atlas (22). Finally, the amount of overlap between the participant’s lesion and the ROIs was calculated using FMRIB Software Library FSL (40).

#### 3.2.2. Probabilistic tractography

HARDI data was acquired using a two-shell high diffusion weighting (b factor = 1000, 2000 s/mm^2^) and 100 gradient (40 mT/m) encoding vectors (50 directions for each shell) alongside 10 non-diffusion weighted (b = 0 s/mm^2^) images, based on the UK BioBank protocol (41) on a Siemens Verio 3T scanner. These parameters maximize diffusion sensitivity and angular contrast while attaining clinically-feasible acquisition times by using a multi-band acceleration factor = 3 (42). Additionally, two b = 0 images with opposite phase encoding directions were acquired prior to the whole acquisition sequence to be used for EPI geometric distortion correction (43, 44). Other HARDI acquisition parameters are: Monopolar spin-echo EPI; 2 mm^3^ isotropic voxel resolution; TR/TE = 3400/94.8 ms; 69 axial slices in the inter-commissural plane for whole-cerebral coverage; FOV = 210 cm; Imaging matrix = 104 × 104; 6/8 phase partial Fourier sampling. Further tractography data processing was done using algorithms from DIPY (45) as implemented in TractoFlow (46). Whole brain probabilistic tractography based on the fiber orientation distribution function (15° angular threshold) was completed and tracts of interest were manually segmented in Mi-Brain (47) using a ROI-based approach described below.

#### 3.2.3. Manual tract segmentation

*In vivo* manual tract dissections of the AF and the FAT were completed using a whole brain tractogram [see (26, 48) for further details on tract segmentation]. Reconstructions of the arcuate fasciculus were based on the three-segment model proposed by Catani et al. (49). The AF long segment was defined by three ROIs placed in the frontal, parietal, and temporal lobes. The frontal ROI was placed anterior to the motor strip, above the insula. The parietal ROI was placed at the arch of the tract in the parietal lobe, in deep white matter. The temporal ROI was placed at the entrance to the temporal lobe, inferior to the Sylvian fissure. The AF anterior segment included only the frontal and parietal ROIs, excluding fibers that passed through the temporal ROI as well. The AF posterior segment included only the parietal and temporal ROIs, excluding fibers that also passed through to the frontal lobe. The frontal aslant tract was defined by one ROI in the IFG and another ROI in the superior SMA. Additional “not-ROIs” were placed to exclude aberrant fibers that did not anatomically or geometrically fit with the tract.

## 4. Results

### 4.1. Language

**Figure 1:**
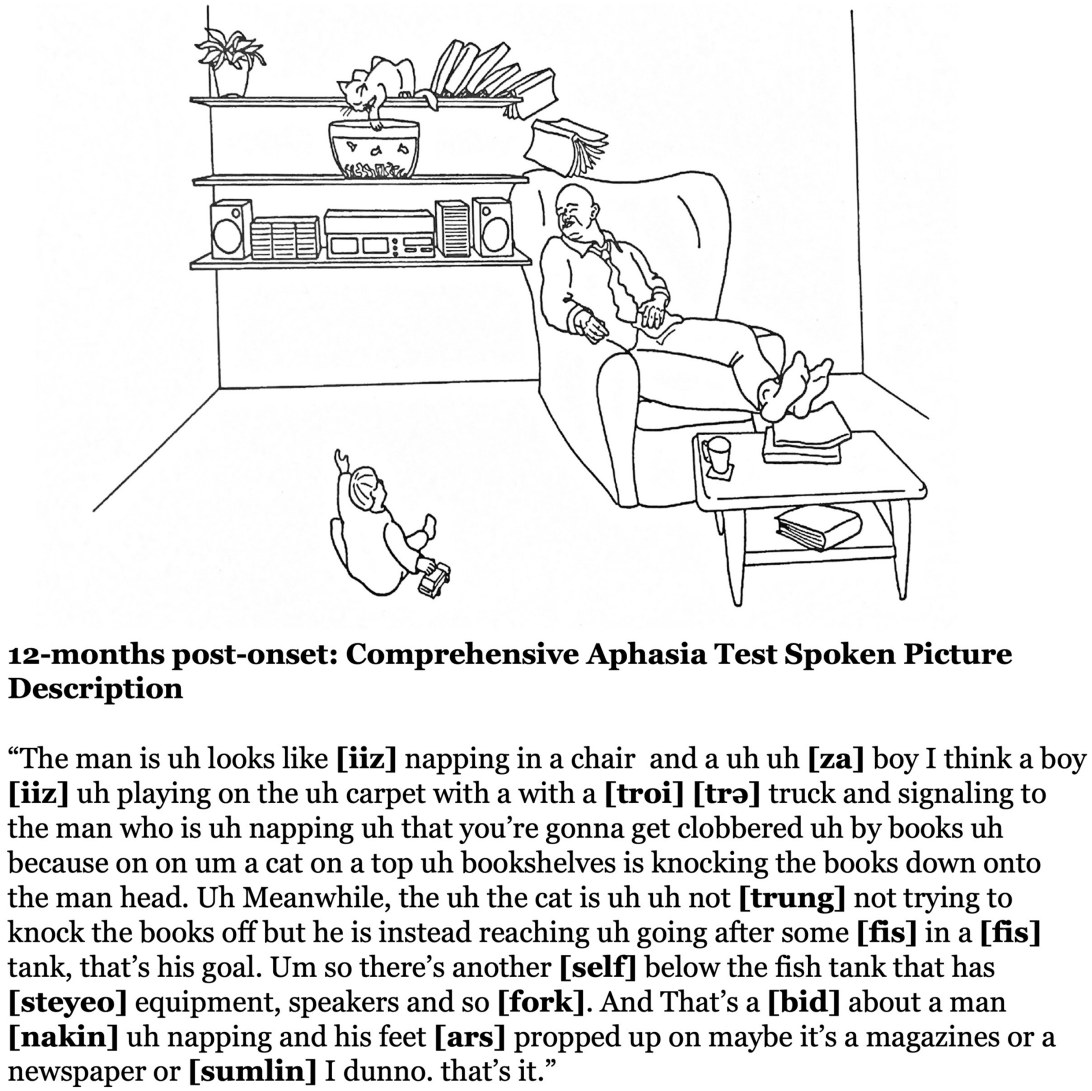
The transcript from the participant’s spoken picture description from the CAT at 12-months post-onset. The participant demonstrates excellent language production free from linguistic errors, yet with a persisting apraxia of speech.

**Figure 2:**
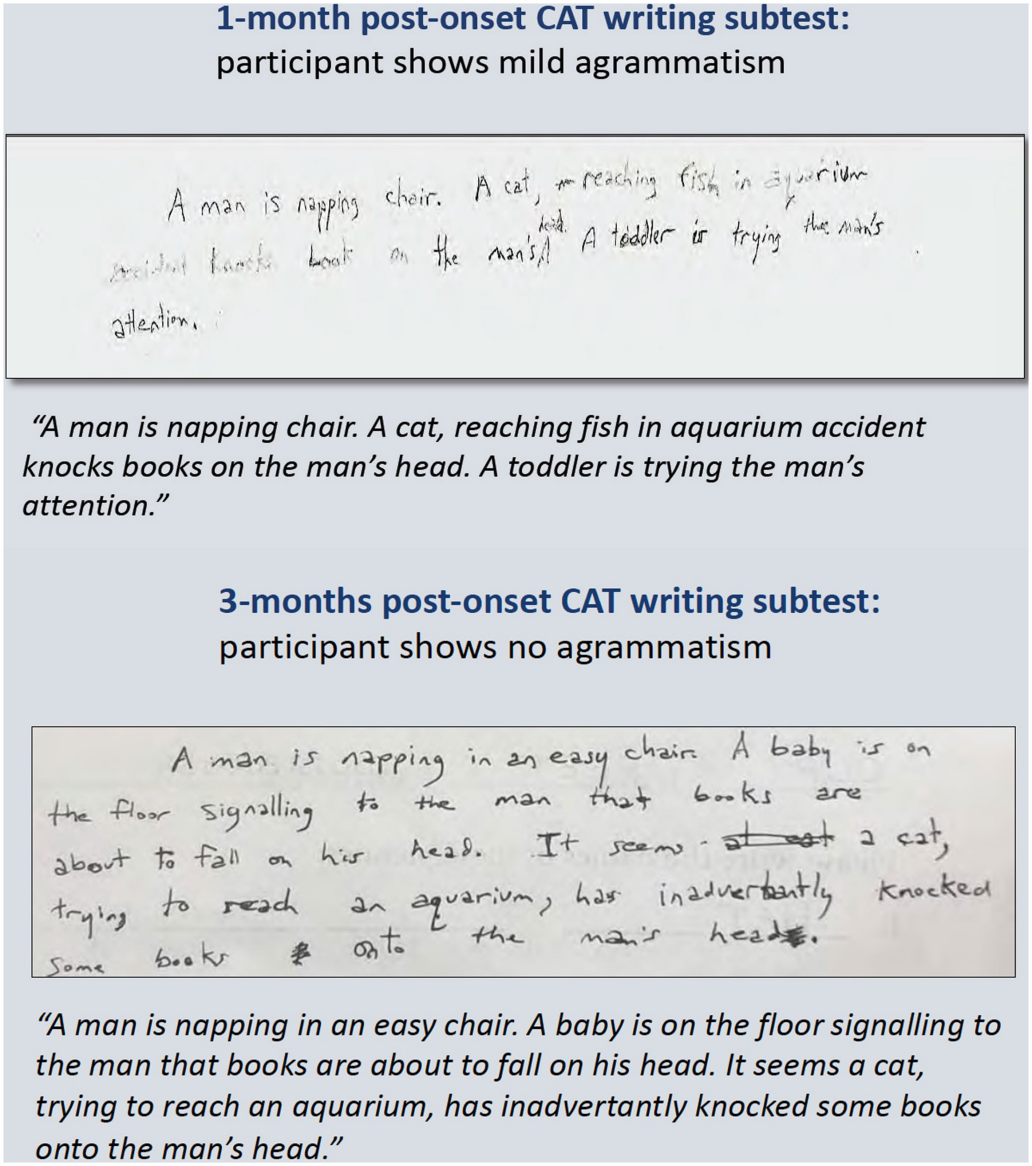
Written picture descriptions from the CAT picture (shown in Figure 1) from 1-month and 3-months post-onset. The agrammatism had resolved by 3-months post-onset.

During the first-year post-stroke, the participant demonstrated a moderately-severe AOS in the absence of persisting language deficits (Table 1; Figure 1). Initially, at 1-month post-onset, he exhibited mild agrammatism which resolved within the first 3 months (Figure 2). From then on, he scored perfectly, or within normal limits, on all CAT subtests except repetition, struggling with longer words and consonant clusters, typical of AOS. He also struggled with the CAT spoken picture description, which is scored in large part on the raw number of information-carrying words produced. Due to difficulty producing speech at a normal rate, his 3-min description was short but within the normal range after 3 months post-onset.

**Table 1:** Results from the CAT, WAB, CYCLE-R, and NNB/NAVS within the first 12 months post-onset.

Comprehensive aphasia test	1-month post-onset	3-months post-onset	12-months post-onset
Cognitive screen (_/38)	35	37	38
Memory (_/20)	19	20	20
Auditory comprehension (_/66)	61	66	66
Written comprehension (_/62)	59	62	62
Repetition (_/74)	58*	68	70
Naming objects (_/48)	48	48	48
Naming actions (_/10)	10	10	10
Spoken picture description	32.5*	45	62
Reading (_/70)	70	70	70
Writing (_/76)	76	76	76
Written picture description	17*	29	37
Western aphasia battery – revised			
Aphasia quotient	94.5	98.2	99.2
Curtiss-Yamada comprehensive language evaluation – receptive (CYCLE-R)			
CYCLE-R raw score	54 (90%)	56 (93%)	58 (96.7%)
Northwestern naming battery/northwestern assessment of verbs and sentences			
Naming nouns	100%	100%	100%
Naming verbs	100%	100%	100%
Sentence production priming	83%	100%	100%

Scores below normal limits are marked in red and with an *. By 3-months, the participant performed within normal limits on all language measures and no longer had aphasia.

### 4.2. Motor speech evaluation

At 1-month post-onset, the participant’s AOS severity was rated as 5 (out of 7) and improved to 4 by his 3-month visit, likely due to training by his SLP at the time to slow his speech. By 12-months post-onset the participant’s AOS score was still considered moderate (4 out of 7). An independent assessment was performed at 12-months post-onset by a licensed speech language pathologist (AR) to confirm the persistence of AOS. At that time, the participant struggled with consonant clusters and conversational speech that demonstrated vowel changes, restricted prosody and intonation, and stress patterns that he perceived as “like a foreign accent,” all typical of AOS. Vowel distortions were likely caused by errors in tongue movements that influenced the vowel sounds around them. Interestingly, the participant produced fluent speech in song. This was demonstrated by his ability to sing familiar songs (‘Happy Birthday’) and to sing his spontaneous thoughts to spontaneous melodies.

On subtests of the Motor Speech Evaluation, the participant demonstrated excellent breath support with no tremors, ruling out other potential motor speech disorders. No articulatory imprecision occurred and there were no consistent articulatory errors typical of any of the dysarthrias (i.e., spastic, flaccid, spastic-flaccid, ataxic, hypokinetic, or hyperkinetic). The SLP also made the assessment that there was no dysarthria based on an oral motor assessment wherein no jaw, lingual, or labial weakness was observed. The SLP further characterized the speech sound errors as “precise” in that articulatory contacts were intact (e.g., lingual elevation to the alveolar ridge for an “s”). However, speech sounds were often produced in the wrong sequence, were inconsistent from production to production, or were influenced by assimilation like /bay-sis-sil/ for bicycle. Sequential motion rates (*puh puh puh*) were relatively intact, as compared to breakdowns in sequencing on alternating motion rates (*puh tuh kuh*), resulting in voicing errors (/*t’ad/, /k’ag/*) and transpositions (*tuh puh kuh*). On multisyllabic word repetitions, the participant demonstrated a similar pattern of voicing, assimilation, and substitution errors as well as syllable deletions on words such as “artillery” (/*truhliy/*) and “catastrophe” (/*kuhtsruhsi/*). Such errors were also abundant in his repetition of sentences and reading of the Grandfather Passage, where rapid transitions between distant articulators proved difficult. Further examples of the different errors made can be found in the Supplementary Appendix.

### 4.3. Neuroimaging findings

**Figure 3:**
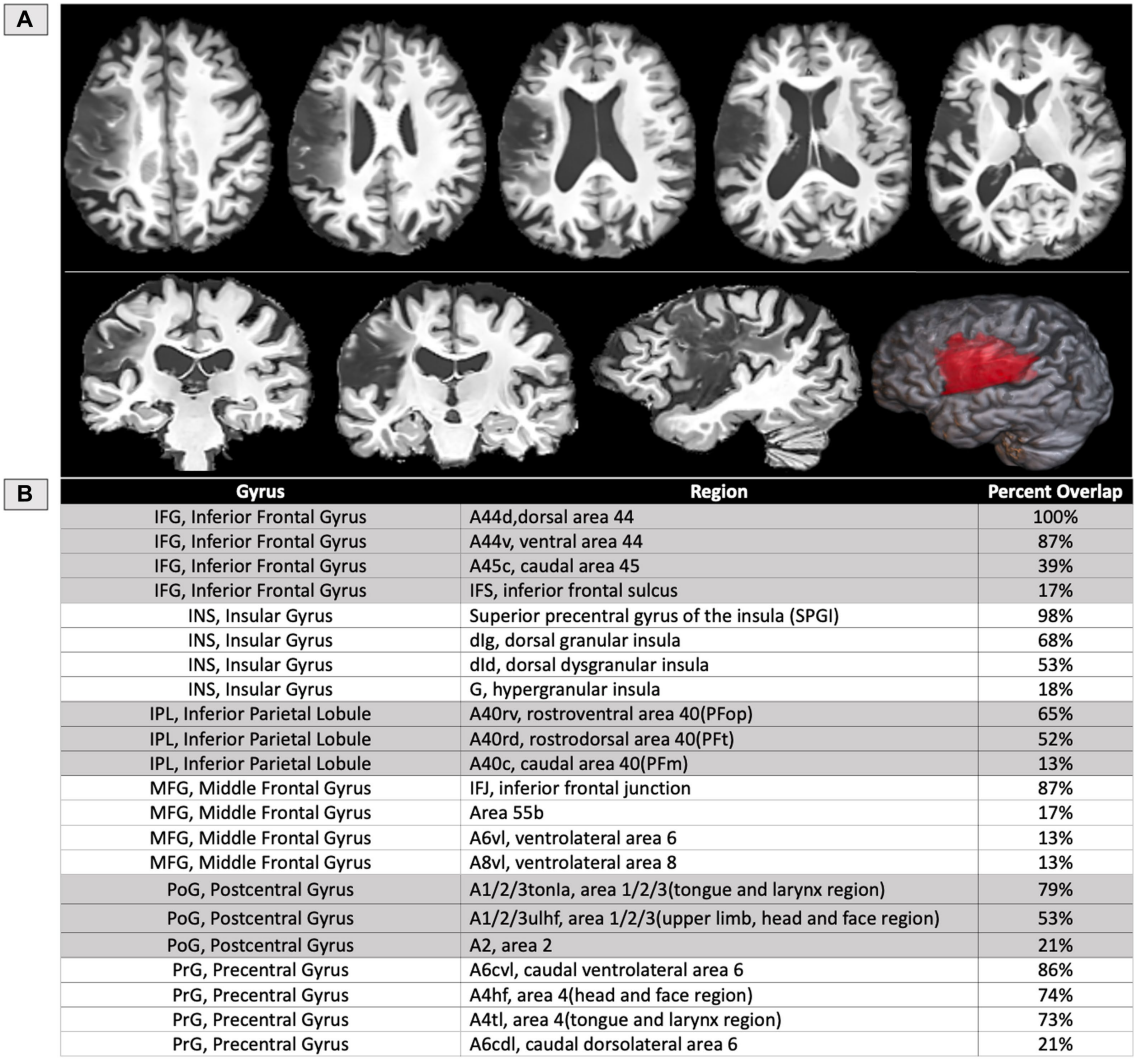
**(A)** T1 structural MRI of the participant’s lesion at 12 months post-onset shown on axial, sagittal, and coronal slices, as well as rendered in 3D. **(B)** Table of the areas damaged at a threshold of 5% or more based on regions of interest from the Brainnetome atlas (39), except for the SPGI, which was constructed based on prior literature (13), and area 55b (22).

The structural scans at 1-year post-onset (Figure 3) indicated a left MCA territory lesion. In the IFG, the most damage was observed in dorsal BA44 (100%), ventral BA44 (87%), caudal BA45 (39%), and the inferior frontal sulcus (IFS) (17%). In the insula, the most damage was observed in the dorsal granular insula (68%), the dorsal dysgranular insula (53%), and the hypergranular insula (18%). The SPGI, a small portion of the anterior insula, was injured at 98%. In the inferior parietal lobule, the most damage was observed in rostroventral BA40 (65%), rostrodorsal BA40 (52%), and caudal BA40 (13%). In the middle frontal gyrus, the most damage was observed in the inferior frontal junction (IFJ) (87%), ventrolateral BA6 (13%), and ventrolateral BA8 (13%). When area 55b was submitted as its own ROI, which does not overlap with the previously listed regions, it was injured at 17%. In the postcentral gyrus, the most damage was observed in the tongue and larynx regions of BA1,2,3 (79%), the upper limb and head and face region of BA1,2,3 (53%), and BA2 (21%). In the precentral gyrus, the most damage was observed in caudal ventrolateral BA6 (86%), the head and face region of BA4 (74%), the tongue and larynx region of BA4 (73%), and caudal dorsolateral BA6 (21%). Percent of damaged tissue in all regions of interest is presented in Figure 3B.

**Figure 4:**
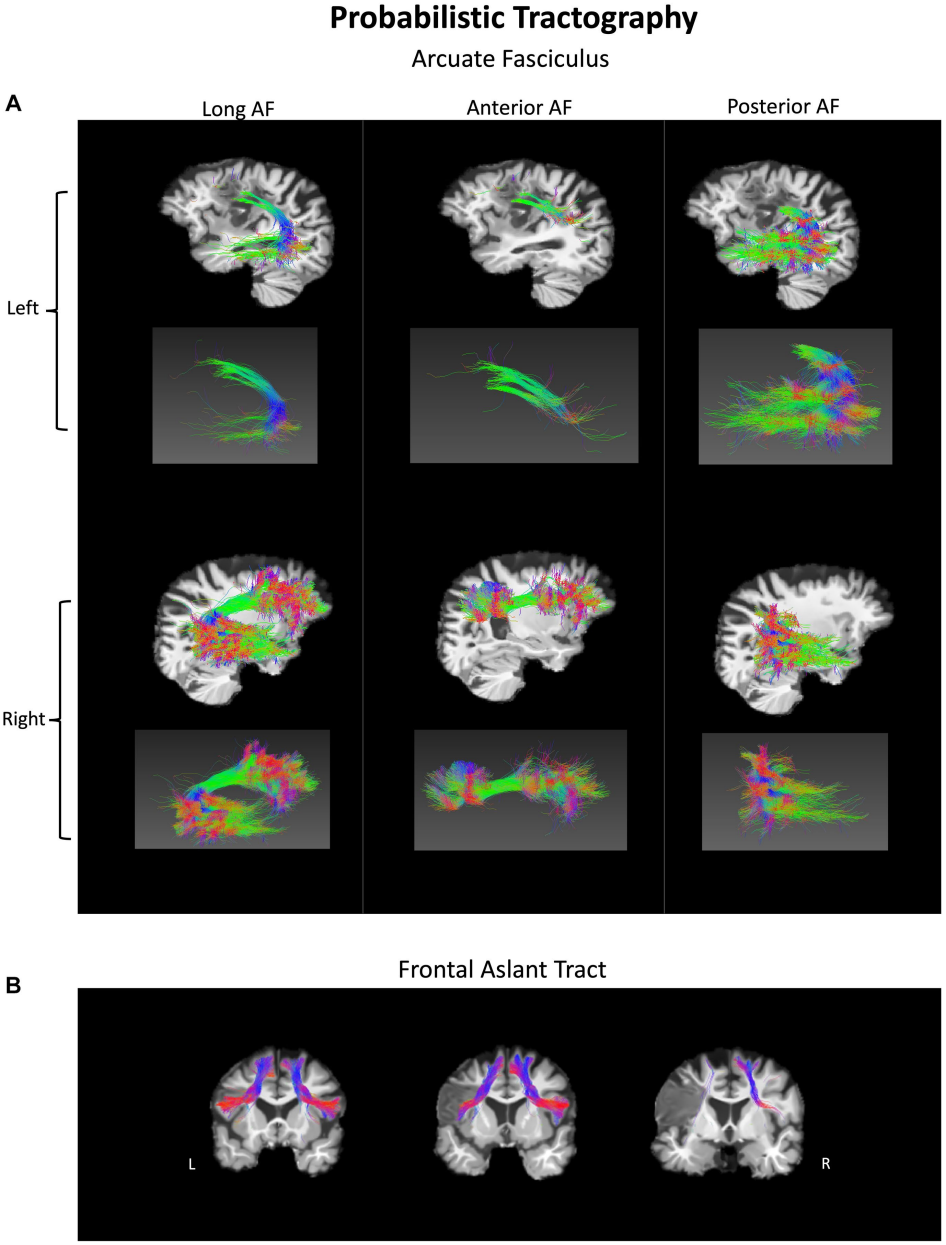
Probabilistic tractography of white matter fiber pathways in a person with pure AOS at 1-year post-onset. **(A)** Arcuate fasciculus (AF) – long, anterior, and posterior components in both hemispheres. **(B)** Frontal aslant tract (FAT) shown in both hemispheres. The lesion disrupts sections of both tracts in the left hemisphere.

The participant’s language scores were unimpaired at 1-year post-onset, leaving only a chronic case of AOS, suggesting that one or more of the lesioned areas are critical for motor speech production. White matter probabilistic tractography was performed at 1-year post-onset. The left AF and FAT have been impacted by the lesion (Figure 4) and show severely decreased fractional anisotropy (FA) and volume (in mL) as compared to their right hemisphere counterparts (Table 2). In particular, the left long AF and the anterior AF are greatly diminished compared to their right hemisphere counterparts, particularly their frontal extensions. At the same time, the left posterior AF was unaffected. The left FAT also shows a reduction in FA and volume as compared to the right FAT. Particularly, the most cortex-reaching fibers of the left FAT are interrupted by the lesion. However, the more medial core of these fibers remained intact.

**Table 2:** Mean tract volume (in mL) and fractional anisotropy values for 3 branches of the arcuate fasciculus (AF) and the frontal aslant tract (FAT) in the left and right hemispheres.

Measure	AF long LH	AF long RH	AF posterior LH	AF posterior RH	AF anterior LH	AF anterior RH	FAT LH	FAT RH
Tract volume (mL)	9.42	66.44	23.63	23.29	4.42	34.46	13.02	28.40
Mean FA	0.26	0.42	0.30	0.37	0.16	0.39	0.30	0.36

## 5. Discussion

The current study presented a pure case of AOS wherein initial language problems were mild but then dissipated, leaving only the motor-speech impairment. This case adds to the existing literature in two ways: (1) This case shows that even in the presence of a large lesion, speech and language can be dissociated and (2) discussed the specific brain areas injured in this participant with pure AOS. In the current study, the participant’s recovery of speech and language were monitored over time. The measures of speech and language were taken at three different time points within the first year, allowing careful monitoring of recovery. This provided the unique opportunity to examine the resolution of certain initial deficits and the persistence of others. The participant’s initial language deficits were not severe, with only a mild agrammatism present that resolved within the first 3 months post-onset. Interestingly, the participant’s performance on motor-speech measures remained impaired, showing only slight improvement due to effortful slowing of speech and rigorous speech therapy (1 to 2 times per week for 45-min sessions in the first-year post-stroke). The reported motor speech disorder did not affect the lexical-semantic or morphosyntactic components of language, which remained intact.

Neuroimaging revealed the most significant amount of damage in dorsal BA44 (100%) and the SPGI (98%), followed by ventral BA44 (87%), the IFJ (87%), caudal ventrolateral BA6 in the precentral gyrus (86%), the tongue and larynx areas in postcentral gyrus (79%), the head and face region of precentral gyrus (74%), the tongue and larynx area of precentral gyrus (73%) and dorsal granular insula (68%) (Figure 3). Notably, more than 90% of the full insula is preserved in this participant, with the insular damage restricted almost exclusively to the superior precentral gyrus of the insula, the small area within the insula previously related to apraxia of speech (13). Also, the current study found possible contributions of fiber pathways to the observed speech deficits such as the FAT and the anterior and long segments of the AF. The left hemisphere FAT was only damaged in extensions to the cortex, suggesting these cortex-reaching projections may also play a role in motor-speech coordination and fluency, as suggested in prior literature (26, 28).

### 5.1. AOS and its definition

Pure AOS is a distinct motor-speech disorder. Historically, AOS has been thought of as a symptom of Broca’s aphasia. This case demonstrates that it can occur and continue in the absence of persisting Broca’s aphasia. A potent factor in the lack of consensus in the AOS literature may be due to inconsistent diagnoses of AOS. In the AOS literature, the time of assessment is critical. Lesions to Broca’s area may cause transient AOS, and though they have their value in understanding the complexity of neural plasticity, studies that are limited to the first 24 h post-onset, may not provide evidence for the causes of long-term pure AOS (10). Other studies involving participants beyond the subacute phase, some even in the chronic phase (2, 11, 13), indicate that persistent AOS can occur with co-existing aphasia. In contrast, there have been cases that show persistent pure AOS (14, 16, 17). In the present study, the MSE was administered at three time points during the first year illustrating a persistent AOS, at first severe, and then moderate. Additionally, an independent speech-language pathologist performed evaluations at 12-months post-onset, confirming the presence of persistent pure AOS, and providing a detailed characterization of the participant’s speech errors (see Supplementary Appendix). This detailed characterization of the participant’s speech and language gives further authority to the claim that the case presented in this study was indeed a case of pure AOS and was not confounded by the presence of other possible speech or language disorders.

### 5.2. Damaged cortical areas in a case of pure AOS

There has long been a dispute about the core lesion site responsible for AOS, whether it be the SPGI, the central operculum, Broca’s area, or Area 55b. In this case, the participant’s left hemisphere infarct caused a lesion encompassing 100% of dorsal BA44 and 98% of the SPGI. Notably, ventral BA44 (87%) was also lesioned, but only 39% of caudal BA45 and 17% of area 55b. The severity of the present case could be impacted by the combination of damage to these regions, as well as surrounding white matter (2). The lesion also affected the inferior parietal lobule’s rostroventral BA40 (65%) and rostrodorsal BA40 (52%). This pattern follows major white matter tracts such as the arcuate fasciculus (35, 50).

It is likely that all these regions play their own unique role in speech articulation. Hillis et al. (10) determined a relationship between Broca’s area and AOS to the exclusion of insular involvement in a cohort of 40 patients with insular damage and 40 without. Notably, the study performed the evaluations within the first 24 h post stroke. Persistent aphasia itself cannot be reliably diagnosed within the first 24 h post-onset, due to edema in the brain as well as other traumatic effects on the patient. Similarly, at 24 h, transient cases of AOS cannot yet be distinguished from persistent ones. However, this criticism touches on an open methodological question regarding the most valid time of assessment to determine brain-behavior relationships. Certainly, there is a great deal to be learned from the symptoms that occur within the first 24 h, as they may reflect brain regions that were involved in speech production. It is also important to understand which functions can reorganize, and which cannot withstand the effects of injury to certain brain areas, as the inability to reorganize a function may indicate a stricter structure–function relationship. This opens the door to more precise questions about how cytoarchitectonic features and electrical activity in specific populations of neurons are related to cognition and behavior. Thus, researchers should not discard studies based on one’s current opinion of validity, but rather, seek to unify them in the broader landscape of research on the brain at different time points pre- and post-stroke. The current study reports damage to the SPGI, a region often contrasted with Broca’s area in the literature with regard to speech praxis. The insula is highly connected to the frontal, temporal and parietal lobes of the brain via white matter pathways. Specifically, the SPGI (98% damaged in our reported case) has connectivity with the superior and inferior frontal lobe (51), which suggests that the inferior frontal lobe and the insula may need to work together to facilitate speech production. Richardson et al. (11) reported 26 cases of chronic left hemisphere stroke resulting in a classification of aphasia with AOS. They suggested that damage to Broca’s area was most reliably predictive of AOS, though the SPGI was not explicitly used as a ROI, rather the entire posterior and anterior regions of the insula were used. In the inferior frontal cortex, the current findings favor a role for dorsal BA44, damaged at 100% and ventral BA44 (87%) over BA45, as only the caudal aspect of BA45 was damaged at 39% in this case of persisting AOS. Richardson and colleagues pointed out that Broca’s area is often referred to as a single area but has sub-regions that may vary in function. For example, BA44 differs cytoarchitectonically from BA45 in cortical layer IV, where they are dysgranular and granular, respectively (52, 53). Prolific post-mortem analysis by Zilles and Amunts (54) revealed average differences between dorsal and ventral BA44 and caudal and rostral BA45. Specifically, dorsal BA44 is richer in serotonin receptors than ventral BA44. Also, rostral BA45 contains relatively higher concentrations of glutamate, GABA, acetylcholine, and serotonin receptors when compared to caudal BA45 (54). There are many sequential and concurrent processing steps involved in the fluent expression of language through speech. Unique aspects of motor speech may be supported by structural features like cell-type and receptor-type, which should be investigated more closely in large-cohort lesion studies in the future.

Lesion overlays as well as VLSM studies, including both acute and chronic participants, indicate the critical role of the left superior precentral gyrus of the insula in AOS for coordinating *complex* articulatory movements (2, 5, 12, 13). Tomaiuolo et al. (14) reported four cases of pure AOS, all with the core lesion located in the left SPGI. Nagao et al. (5) reported transient AOS resulting from a small focal lesion to the SPGI, further illustrating the unique connection between this small brain region and the disorder. The Brainnetome atlas overlap (39) showed damage to dorsal granular insula (68%) and dorsal dysgranular insula (53%), which are both cytoarchitectonically defined regions that encompass the SPGI. Importantly, the SPGI does not appear to be involved in producing simple speech movements (as in quickly repeating ‘banana’ 5 times), something that is easier for patients with AOS than more complex articulatory movements. Only the more complex movements that involve rapid transitions between distant places of articulation – particularly words and phrases with consonant clusters (‘graffiti,’ ‘gloating’) – pose a problem for those with AOS and SPGI lesions.

Graff-Radford et al. (17) showed 5 cases of pure AOS, evaluated at a median of 3 days post-stroke, and related AOS to damage to the left premotor and motor cortices. Though most of their cases had orofacial weakness or right-hand weakness, the cases reported had persisting motor speech deficits that were classified as AOS. Additionally, the time frame of evaluation (around 3 days post-stroke), increases the likelihood that edema affected the observed behavioral symptoms. Nonetheless, the left precentral gyrus contains the motor face area, which is likely involved in executing motor speech commands during speaking and, if damaged, could exacerbate the symptoms of AOS. Itabashi et al. (16), also found in a VLSM analysis of 136 patients with left MCA stroke that the posterior wall of the left precentral gyrus was the center of AOS symptoms. In total, their cohort contained 7 patients with pure AOS and 15 patients with AOS and concomitant aphasia. The motor strip (precentral gyrus), that controls motor movements, is directly posterior to BA44. Importantly, in the current case, the area that is damaged encompasses the motor face area, which is key for executing the movements needed in speech articulation. Specifically, the most damage was observed in caudal ventrolateral BA6 (86%), the head and face region of BA4 (74%), the tongue and larynx region of BA4 (73%), and caudal dorsolateral BA6 (21%). Frontal areas (IFG, MFG) that abut the precentral gyrus may interface with the motor strip and deeper structures, like the insula, to execute articulatory movements.

Additionally, we find that the participant’s lesion overlaps with 17% of area 55b and 87% of the inferior frontal junction (IFJ). Area 55b is an emerging region of interest that overlaps with the posterior MFG, which has connectivity with the arcuate fasciculus/superior longitudinal fasciculus. Chang et al. (23) published a case of a pure speech production deficit after surgical resection of area 55b (23). Our findings suggest that 55b could be involved in the deficits seen in AOS, though only 17% is impacted by the lesion in this case. However, the surgical resection in Chang et al. (23) aimed to remove a grade III astrocytoma. Tumors cause more gradual change than strokes, and often allow for reorganization of neural function. While 55b may indeed play a role in speech production, we must be mindful of how reorganization due to tumor-growth remodels the landscape of speech and language organization.

### 5.3. Damaged fiber pathways in a case of pure AOS

In the current study, we identify interruptions of the left FAT’s cortex-reaching fibers as well as the left long segment and anterior segment of the AF. The left FAT has been associated in previous work with fluency (26), and this case of pure AOS suggests a more specific role in speech coordination. Our findings are also congruent with Valls Carbo et al. (28), who showed abnormal diffusion in white matter projections from the SMA to inferior frontal cortex via the FAT in 36 cases of PPAOS (28). Both the left FAT and the left anterior AF interact with frontal language areas, such as the motor face area and the IFG. The left anterior AF connects frontal and parietal regions. Interestingly, another form of apraxia, limb apraxia, can be induced by parietal damage, which is markedly different from AOS. Perhaps the frontal and parietal regions work in tandem to facilitate different aspects of motor coordination, with more frontal regions dedicated to speech coordination and more parietal regions dedicated to limb coordination. Notably, the tracts interrupted by the lesion show a reduction in FA, consistent with previous findings in aphasia (48, 50). High-resolution tractography is uncommon in the AOS literature, and especially rare for pure AOS. The current study provides a unique opportunity to combine lesion analysis with the investigation of the underlying white matter pathways.

### 5.4. Limitations

This case of pure AOS, while informative, is subject to the inherent limitations of a case study. Given that this is a single case, the results may not be generalizable due to the high individual variability of the human brain. The large lesion further complicates the interpretation of observed speech deficits and their relation to any specific area. The ROI-based analysis employed in this study offers only one approach to segmenting potentially involved brain regions and might oversimplify the complex interplay of different brain areas in causing AOS. Indeed, the true neural basis of AOS might not be fully captured by the defined ROIs, possibly involving smaller or different areas. Moreover, while we discuss the damage to the SPGI and BA44, we do not imply that these regions are necessarily more crucial for AOS than other damaged regions, such as the precentral gyrus or Broca’s area. We sought to provide a detailed interpretation of the damage to various subregions implicated in the previous literature. Acknowledging these limitations, the data will be made available upon request for other analytical approaches and inclusion in larger cohort studies.

## 6. Conclusion and future work

In this case study, the participant had suffered injury to multiple regions involved in speech production and articulation. His lesion involved 98% of the SPGI, with additional involvement of three other areas that have been cited in the previous literature: BA44, the motor face area, and area 55b. Surrounding white matter tracts were also implicated. Recovery from AOS is a long and difficult process. Speech-language therapy can decrease the severity, but an individual diagnosed after the acute phase is likely to experience the symptoms of AOS long after the initial stroke. Cases of persisting AOS speak to the fact that certain speech production mechanisms are hardwired, meaning that complete functional reorganization is precluded. People with chronic AOS are not completely bereft of speech, but fluent coordination of articulation is permanently altered. Speech production, which includes the coordination of complex articulatory movements, requires the collaboration of multiple brain regions with differing contributions. It is of critical importance that the field continues to work to understand the underlying mechanisms that cause AOS, with the end goal of improving the lives of the individuals affected by it. Pure cases such as this provide a unique opportunity to identify mechanisms that, when damaged, result in specific disorders.

## Data availability statement

The raw data supporting the conclusions of this article will be made available by the authors, following HIPAA guidelines.

## Ethics statement

The studies involving human participants were reviewed and approved by the University of California Berkeley Committee for Protection of Human Subjects. The patient/participant provided their written informed consent to participate in this study.

## Author contributions

AP collected the data, analyzed it, and drafted the manuscript. ND and MI designed neuromaging protocols, and also participated in the data collection, analysis and drafting the manuscript. AR performed a detailed speech assessment and drafted the parts of the manuscript that discuss the behavioral performance on speech and language assessments. All authors contributed to the article and approved the submitted version.

## Funding

This work was supported by the National Institute on Deafness and Other Communication Disorders (R01DC016345 and R21DC021042).

## Conflict of interest

The authors declare that the research was conducted in the absence of any commercial or financial relationships that could be construed as a potential conflict of interest.

## Publisher’s note

All claims expressed in this article are solely those of the authors and do not necessarily represent those of their affiliated organizations, or those of the publisher, the editors and the reviewers. Any product that may be evaluated in this article, or claim that may be made by its manufacturer, is not guaranteed or endorsed by the publisher.

## Footnotes

^1^These subtests are particularly difficult for people with AOS (2).
