# Supplementary material for: A case of pure apraxia of speech after left hemisphere stroke: behavioral findings and neural correlates
Source: Front Neurol. 2023 Jul 27;14:1187399. doi: 10.3389/fneur.2023.1187399 (PMC10421996; doi:10.3389/fneur.2023.1187399)
Supplement: Supplementary file 1 [file Table_1.docx]

| Voicing Errors in AMRs | d for t and g for k |
| --- | --- |
| Transposition Errors in AMRs | t^p^k^ for p^t^k^ |
| Transposition Errors in Connected Speech | Fridgreator for refrigerator |
| Vowel Errors in SMRs/AMRs | ^ becomes ae  in productions with /k/, possibly influenced by glottal k and the slight lingual elevation for posterior lingual contact moves the lax vowel ^ to ae |
| Vowel Errors in Connected Speech | Snake for sneak; vowel change from /i/ --->/ae/; butiful/beautiful; deletion of i prior to oo (u) so could be omission instead of vowel change |
| Syllable Deletion | /ætr^li/ for artillery |
| Substitutions | Most prevalent and consistent pattern observed is placement shifts from palatal to alveolars: wasd/washed, wisd/wished, see/shee, same/shame |
| Assimilation Errors | Bisisul for bicycle, begiging for beginning, slowless for slowness, |
| Anticipatory Errors | Caztazrase for catastrophe; insertion of /z/  in first syllable in anticipation of 2nd syllable |
| Inconsistent Errors | winkon, winton for winston; on repetitions he achieves close approximations to eventual target “winston”. |
| Abnormal Prosodic Features | monotone, restrictive intonational pattern, emphasis achieved with loudness v. prosodic shifts. |

**Appendix Table.** Findings from the motor speech assessment by a speech language pathologist trained in the assessment and treatment of AOS (AR). Descriptions of motor speech errors as well as examples are indicated in the table.
